# Cost-Effectiveness of Pelvic Floor Reconstruction Using Biological Mesh After Extralevator Abdominoperineal Resection for Rectal Cancer

**DOI:** 10.1097/DCR.0000000000004326

**Published:** 2026-06-15

**Authors:** Rudolf van den Berg, Gijsbert D. Musters, Cornelis Verhoef, Sarah Sharabiany, Roel Hompes, Baljit Singh, Sanjay S. Chaudhri, Anna A.W. van Geloven, Ronald J.C.L.M. Vuylsteke, Johannes H.W. de Wilt, Jeroen W.A. Leijtens, Maarten Vermaas, Joost Rothbarth, Jacobus W.A. Burger, Bas Lamme, Anthony van de Ven, Hedwig M. Blommestein, Pieter J. Tanis

**Affiliations:** 1 Department of Surgery, Erasmus University Medical Center, Rotterdam, the Netherlands; 2 Department of Surgery, Zaans Medical Center, Zaandam, the Netherlands; 3 Department of Surgery, Amsterdam UMC location University of Amsterdam, Amsterdam, the Netherlands; 4 Cancer Centre Amsterdam, Treatment and Quality of Life, Amsterdam, the Netherlands; 5 Department of Surgery, Amsterdam UMC location VU University Medical Center, Amsterdam, the Netherlands; 6 Department of Surgery, University Hospitals Leicester, Leicester, United Kingdom; 7 Department of Surgery, Tergooi Medical Centre, Hilversum, the Netherlands; 8 Department of Surgery, Spaarne Gasthuis, Haarlem, the Netherlands; 9 Department of Surgery, Radboud University Medical Centre, Nijmegen, the Netherlands; 10 Department of Surgery, St. Laurentius Hospital, Roermond, the Netherlands; 11 Department of Surgery, IJsselland Hospital, Capelle aan de IJssel, the Netherlands; 12 Department of Surgery, Catharina Hospital, Eindhoven, the Netherlands; 13 Department of Surgery, Albert Schweitzer Hospital, Dordrecht, the Netherlands; 14 Department of Surgery, Flevo Hospital, Almere, the Netherlands; 15 Health Technology Assessment, Erasmus School of Health Policy and Management, Erasmus University Rotterdam, Rotterdam, the Netherlands

**Keywords:** Abdominoperineal resection, Cost-effectiveness, Health economics, Perineal reconstruction, Postoperative complications, Quality-adjusted life years, Rectal neoplasms, Surgical mesh, Wound closure techniques, Wound healing

## Abstract

**BACKGROUND:**

Perineal complications are a major source of morbidity after extralevator abdominoperineal resection for low rectal cancer, especially after neoadjuvant (chemo)radiotherapy. Biological mesh reconstruction can reduce perineal hernia formation, but its cost-effectiveness remains unclear.

**OBJECTIVE:**

To evaluate the cost-effectiveness of biological mesh reconstruction compared with primary perineal closure after extralevator abdominoperineal resection in patients with low rectal cancer.

**DESIGN:**

This study was designed as a long-term economic evaluation conducted alongside a multicenter, parallel-group, randomized controlled trial.

**SETTINGS:**

Eleven hospitals in the Netherlands and 1 hospital in the United Kingdom, including nonteaching, teaching, and university centers.

**PATIENTS:**

Adult patients with primary low rectal cancer were enrolled in the study.

**INTERVENTIONS:**

Primary layered perineal closure versus pelvic floor reconstruction using a biological, non–cross-linked porcine dermal mesh.

**MAIN OUTCOME MEASURES:**

Total health care costs from a health care payer perspective, incorporating surgery, hospitalization, outpatient care, wound management, readmissions, and reinterventions; and patient health outcomes measured as quality-adjusted life years calculated from standardized health utility scores.

**RESULTS:**

Of 104 randomly assigned patients, 101 were included in the final analysis (53 primary closures, 48 biological mesh). Mean total health care costs were higher in the mesh group (€57,991 vs €56,232; mean difference €1571; 95% CI, –€9840 to €12,981; *p* = 0.785). Perineal complications significantly reduced quality of life, but wound complication rates and quality-of-life gains were modest for mesh, with no significant differences between groups over time.

**LIMITATIONS:**

The study sample size limited the precision of cost and subgroup estimates.

**CONCLUSIONS:**

Biological mesh reconstruction increases procedural and hospitalization costs without improving long-term patient outcomes. Routine use cannot be recommended from a cost-effectiveness perspective. See **Video Abstract**.

**COSTO-EFECTIVIDAD DE LA RECONSTRUCCIÓN DEL SUELO PÉLVICO MEDIANTE MALLA BIOLÓGICA TRAS LA RESECCIÓN ABDOMINOPERINEAL EXTRALEVADORA PARA EL CÁNCER RECTAL:**

**ANTECEDENTES:**

Las complicaciones perineales constituyen una fuente importante de morbilidad tras la resección abdominoperineal extralevadora para el cáncer de recto bajo, especialmente después de recibir (quimio)radioterapia neoadyuvante. La reconstrucción con malla biológica puede reducir la formación de hernias perineales, pero su rentabilidad sigue sin estar clara.

**OBJETIVO:**

Evaluar la rentabilidad de la reconstrucción con malla biológica en comparación con el cierre perineal primario tras la resección abdominoperineal extralevadora en pacientes con cáncer de recto bajo.

**DISEÑO:**

Evaluación económica a largo plazo realizada de forma concomitante a un ensayo controlado aleatorizado, multicéntrico y de grupos paralelos.

**ÁMBITO:**

Once hospitales en los Países Bajos y un hospital en el Reino Unido, incluyendo centros no docentes, docentes y universitarios.

**PACIENTES:**

Pacientes adultos con cáncer de recto bajo primario.

**INTERVENCIONES:**

Cierre perineal primario por planos frente a reconstrucción del suelo pélvico mediante una malla dérmica porcina biológica no reticulada.

**PRINCIPALES MEDIDAS DE RESULTADO:**

Costes sanitarios totales desde la perspectiva del pagador sanitario, que incluyen cirugía, hospitalización, atención ambulatoria, manejo de heridas, reingresos y reintervenciones; y resultados de salud del paciente, medidos en años de vida ajustados por calidad (AVAC), calculados a partir de puntuaciones estandarizadas de utilidad en salud.

**RESULTADOS:**

De los 104 pacientes aleatorizados, 101 fueron incluidos en el análisis final (53 en el grupo de cierre primario y 48 en el de malla biológica). Los costes sanitarios totales medios fueron superiores en el grupo de la malla (57.991 € frente a 56.232 €; diferencia media: 1.571 €; IC del 95 %: -9.840 € a 12.981 €; p = 0,785). Las complicaciones perineales redujeron significativamente la calidad de vida; sin embargo, las tasas de complicaciones de la herida y las mejoras en la calidad de vida obtenidas con la malla fueron modestas, sin observarse diferencias significativas entre los grupos a lo largo del tiempo.

**LIMITACIONES:**

El tamaño de la muestra del estudio limitó la precisión de las estimaciones de costes y de los subgrupos.

**CONCLUSIONES:**

La reconstrucción con malla biológica incrementa los costes del procedimiento y de la hospitalización sin mejorar los resultados de salud del paciente a largo plazo. Por tanto, desde una perspectiva de rentabilidad, no se puede recomendar su uso rutinario. *(AI-generated translation*)

Perineal wound complications occur commonly after abdominoperineal resection (APR) for low rectal cancer, resulting in considerable patient morbidity and increased health care resource use.^1^ These complications, such as perineal wound infection, dehiscence, and pelvic abscesses, often necessitate prolonged wound care that can extend for months, sometimes culminating in chronic perineal sinuses or fistulas across a year.^2^ The associated pain, difficulties with sitting and walking, and ongoing wound management requirements adversely affect patients’ quality of life (QoL).^3^ In addition, APR poses a considerable risk of perineal hernia, which further impacts QoL.^4,5^ Although only a minority of patients with colorectal cancer require APR, the high overall incidence of colorectal cancer in Western countries means that perineal wound complications and hernia formation still represent an important clinical and economic challenge.^6,7^

Surgical advances, including the extralevator APR (eAPR) approach, and preoperative (chemo)radiotherapy have improved oncological outcomes.^8^ However, these benefits have come at the cost of increased perineal wound morbidity. The eAPR technique, involving wider resection with levator muscle excision, results in a larger pelvic floor defect, which presents challenges for effective closure. Standard closure involves primary layered suturing of perineal tissues. Biological meshes derived from acellular porcine dermis have been introduced as an adjunct to reconstruct the pelvic floor.^9,10^

Despite increasing clinical use, the evidence supporting biological mesh reconstruction after eAPR remains limited. To date, no consensus exists on the optimal method for perineal closure regarding both clinical effectiveness and cost-effectiveness. The BIOPEX randomized controlled trial was designed to evaluate the clinical efficacy of biological mesh reconstruction compared with primary perineal closure after eAPR in patients with low rectal cancer who had received preoperative (chemo)radiotherapy.^11,12^

In the present economic evaluation conducted alongside the BIOPEX trial, we aimed to assess the cost-effectiveness of pelvic floor reconstruction using biological mesh relative to primary perineal closure, focusing on health care costs and quality-adjusted life years (QALYs). This evaluation provides essential evidence to guide surgeons, patients, and health care policymakers in resource allocation decisions for this high-risk patient population.

## MATERIALS AND METHODS

### Study Design and Setting

This cost-effectiveness analysis was based on the BIOPEX trial, a multicenter, parallel-group, single-blinded randomized controlled trial conducted in 1 nonteaching hospital, 6 teaching hospitals, and 4 university hospitals in the Netherlands, as well as 1 university hospital in the United Kingdom.^12^ Patients and perineal wound assessors were blinded to treatment allocation. The design and methodology of the BIOPEX trial have been previously described.^13^ In short, eligible patients underwent eAPR for primary rectal cancer after neoadjuvant radiotherapy and were randomly assigned in a 1:1 ratio to either primary layered perineal closure or pelvic floor reconstruction with a biological mesh followed by layered perineal closure. The trial protocol was approved by institutional review boards at all participating sites, and written informed consent was obtained from all patients. The trial is registered at ClinicalTrials.gov (NCT01927497).

### Economic Evaluation

An economic evaluation was conducted alongside the BIOPEX trial from the perspective of the Dutch health care system, supplemented with informal care costs. Costs were reported in 2025 euros and included procedural costs, hospitalization, wound-related care, postdischarge care, and costs related to reinterventions. Resource use data were collected prospectively during the trial’s original follow-up and long-term follow-up.^11,12^ The time horizon for analyses of the economic evaluation was 5 years. Cost valuations were based on the updated Dutch Costing Manual and price lists from participating hospitals. Costs recorded in earlier years were adjusted to 2025 euros using the Dutch Costing Manual. As cost data from the UK center could not be obtained, UK procedures were valued using Dutch unit prices.

### Procedural Costs

Perineal wound closure techniques differed between study arms. In the standard treatment arm, the perineal wound was closed in layers. The ischioanal and subcutaneous fat were approximated using interrupted Vicryl sutures (Ethicon Inc, Johnson & Johnson, New Brunswick, NJ), and the skin was closed with interrupted sutures at the discretion of the operating surgeon. The decision to place an abdominal and/or perineal drain was left to the local surgeon’s judgment. In the experimental arm, pelvic floor reconstruction was performed using a 6 × 10 cm acellular, non–cross-linked biological mesh derived from porcine dermis (Strattice, LifeCell, an Acelity Company, Branchburg, NJ). The mesh was secured using interrupted Prolene or polydioxanone sutures (Ethicon Inc, Johnson & Johnson). Posteriorly, it was anchored to either side of the coccyx or sacrum; laterally, it was attached to the remnants of the levator ani muscles; anteriorly, it was fixed to the transverse perineal muscles. A suction drain was placed over the mesh, and the ischioanal and subcutaneous fat and skin were closed in the same manner as in the control group.

Cost differences in the techniques during surgery stemmed primarily from the costs of the biological mesh and increased operative time. Mesh unit prices were obtained from the manufacturers’ price list. Operating time (incision-to-closure in minutes) was extracted from the trial database and monetized using operating theater cost per minute from the Dutch Costing Manual.^14^ Total eAPR procedural costs were estimated using unit prices from participating centers.

### Hospitalization Costs

Length of stay before and after surgery, as well as days in the intensive care unit (ICU), were obtained from patient records and valued using Dutch national reference prices.^14^ ICU costs were calculated separately.

### Posthospitalization Care

Discharge destination and postdischarge care (e.g., rehabilitation, formal and informal home care) were recorded during the study. Care duration was valued using cost estimates stratified by intensity of care.^14^

### Wound-Related Care

Data on the type, frequency, and duration of wound care (e.g., dressing changes, outpatient visits, and negative pressure wound therapy (NPWT)) were obtained from the trial scores. The prices of consumables (e.g., bandages, incontinence materials) were averaged across 3 representative institutions (Erasmus MC, Zaans MC, and MEDIQ). Estimates of material use per dressing were based on expert input from clinical staff.

### Reinterventions and Rehospitalizations

Surgical and radiological interventions related to perineal hernia or wound complications were identified via the trial database, and rehospitalization costs were calculated using standard hospital tariffs for stay on the ward and ICU.^14^ Perineal hernia was defined as either a clinically evident perineal bulge confirmed on physical examination or radiological evidence of abdominal contents descending below the pubococcygeal line. CT imaging protocols varied by center but were performed per local follow-up schedules or when clinically indicated. Some patients signed informed consent for pelvic MRI without dynamic imaging after 3 to 5 years of follow-up for long-term evaluation.

### Health-Related QoL

Health-Related QoL (HRQoL) was assessed using the EuroQoL five-dimension, five-level questionnaire (EQ-5D-5L) and EQ-visual analog scale (VAS) at baseline, 1, 3, 6, and 12 months postoperatively. Researchers independent of the operating surgeons distributed and collected questionnaires with nonresponders receiving up to 2 reminders. Health utility values were derived using the Dutch EQ-5D-5L tariff by Versteegh et al.^15^ These utilities supported QALY calculations in secondary analyses.^15^

### Statistical Analysis

Categorical variables were summarized as frequencies and percentages; continuous variables as means ± SD or medians with interquartile ranges (IQRs), depending on distribution. Fisher exact probability test, Student *t*-test, or Wilcoxon rank-sum test was used for group comparisons, as appropriate.

Cost data were summarized as means, medians, and 95% CIs. HRQoL data were analyzed using beta-regression models with random intercepts to account for within-subject correlation, adjusting for treatment group.

Missing data were addressed using multiple imputation via predictive mean matching (20 imputations, 30 iterations). Imputation models included age, randomization group, presence of presacral abscess, and Southampton Wound Scores at multiple time points. For deceased patients, cost accumulation was censored at death and utilities after death were set at zero.

In each imputed data set, 10,000 bootstrap resamples were drawn to estimate mean cost differences. Rubin’s rules were used to pool estimates. Welch’s *t* tests were applied to compare per-patient mean costs. We performed deterministic 1-way and 2-way sensitivity analyses to test the robustness of the base-case cost results to plausible changes in the 2 key drivers of procedural cost: mesh unit price and extra operating room (OR) time associated with mesh placement. The mesh price varied from 50% to 150% of the base price. Extra OR time varied from 50% to 150% of the base extra time. For the 2-way analysis, we evaluated a 20 × 20 grid crossing the mesh price and extra OR time ranges and visualized the results as a heatmap. All analyses were conducted using R (version 4.5.0) with the “mice,” “boot,” and “stats” packages.

## RESULTS

In the BIOPEX trial, 104 patients were randomly assigned, of whom 54 were assigned to primary perineal wound closure and 50 to biological mesh closure. After randomization, 1 patient in the primary perineal closure group died before surgery, and 2 patients in the biological mesh closure group underwent a sphincter-preserving procedure instead of eAPR. As these 3 patients were not eligible for evaluation of the primary end point, they were excluded from further analysis, leaving 53 patients in the standard arm and 48 in the intervention arm available for cost-effective analyses in a modified intention-to-treat population. Table 1 presents the baseline characteristics of the study population. The overall median follow-up duration postoperatively was 4.7 years (IQR, 3.4–5.1), with comparable follow-up times in the primary closure group and the biological mesh group (*p* = 0.380).

**TABLE 1.** Baseline characteristics of the included patients

*Characteristic*	*Primary wound closure*	*Biological mesh closure*
	*(N = 53*)	*(N = 48*)
Hospital type		
Nonteaching	8% (4/53)	15% (7/48)
Teaching	55% (29/53)	44% (21/48)
University	38% (20/53)	42% (20/48)
Sex		
Female	26% (14/53)	25% (12/48)
Male	74% (39/53)	75% (36/48)
Age, y, mean (SD)	64 (12)	65 (12)
BMI, mean (SD)	26 (3)	26 (5)
Weight loss and nutritional optimization		
>10% weight loss before surgery	9% (5/53)	6% (3/48)
ASA classification		
I	70% (37/53)	60% (29/48)
II	28% (15/53)	35% (17/48)
III	2% (1/53)	4% (2/48)
Current smoker	11% (6/53)	6% (3/48)
Comorbidities		
Diabetes	8% (4/53)	10% (5/48)
Respiratory disease	9% (5/53)	8% (4/48)
Cardiovascular disease	11% (6/53)	21% (10/48)
Vascular disease	2% (1/53)	4% (2/48)
Immunosuppressant medication	4% (2/53)	0% (0/48)
Diverting stoma due to obstruction	8% (4/50)	8% (4/48)
Distance to anorectal junction on MRI, median (IQR)	1 (0–3)	2 (0–3)
Preoperative radiotherapy		
Short course	19% (10/53)	21% (10/48)
Long course (chemo)radiotherapy	79% (42/53)	79% (38/48)

IQR = interquartile range.

### Primary Closure and Biological Mesh

The mean procedural costs without hospitalization were calculated at €37,192 (see Supplemental Table 1 at https://links.lww.com/DCR/C632). According to the Dutch Costing Manual, OR time was valued at €11.09 per minute.^14^ Mean duration of eAPR with mesh closure was 52 minutes longer (95% CI, 21–82) compared to eAPR with primary closure. The overall additional costs of the mesh were estimated at €2377 euros (6.4% increase in the estimated procedural costs; see Supplemental Table 1 at https://links.lww.com/DCR/C632). The combined mean cost of inpatient and intensive care hospitalization for the primary procedure was €10,035 (95% CI, 9367–10,703) in the primary closure group and €11,729 (95% CI, 10,918–12,540) in the mesh group.

### Wound Care Costs

A total of 12 patients (23%) in the primary closure group and 14 patients (29%) in the mesh group required home-based wound care. The mean duration of total home care for patients receiving care was 33 hours (SD 85) and 19 hours (SD 38) in the respective groups. Among those who received home care, the mean duration was 22 weeks (95% CI, 3–40) in the primary closure group versus 33 weeks (95% CI, 12–54) in the mesh group, and mean cost of home care was €1619 (95% CI, 257–2980) and €2486 (95% CI, 915–4057), respectively. The mean total duration of wound dressing or diaper use was 95 weeks (SD 129) in the primary closure group and 59 weeks (SD 75) in the mesh group. NPWT was administered to 2 patients (4%) in each of the 2 groups. The mean duration of NPWT was 7 days in both groups, with corresponding mean costs of €220.

### Postprocedure Intervention and Rehospitalization Costs

Ten patients in the primary closure group (19%) and 5 patients in the mesh group (10%) required a reoperation. The mean total duration of readmission was 2.8 days in the primary closure group and 1.5 days in the mesh group. The estimated costs of postprocedure complications were €16,048 for perineal hernia repair, €2672 for surgical abscess drainage, €1050 for radiological abscess drainage, and €14,569 for ileus surgery. The timing of postprocedure interventions and readmissions is provided in Supplemental Table 2 at https://links.lww.com/DCR/C633.

### Quality of Life

At baseline, the mean and median VAS scores were 78.6 (95% CI, 74.4–82.8) and 80.0 (IQR, 70.0–90.0) in the primary closure group and 75.2 (95% CI, 70.0–80.3) and 80.0 (IQR, 70.0–85.0) in the mesh group. The corresponding mean and median EQ-5D-5L scores were 83.1 (95% CI, 79.4–86.9) and 84.5 (IQR, 77.5–87.6) for the primary closure group and 84.7 (95% CI, 80.2–89.3) and 84.5 (IQR, 80.4–100.0) for the mesh group.

At 1-year follow-up, the mean and median VAS scores were 81.7 (95% CI, 77.0–86.5) and 85.0 (IQR, 77.5–90.0) for the primary closure group and 76.1 (95% CI, 71.5–80.7) and 80.0 (IQR, 70.0–90.0) for the mesh group. The mean and median EQ-5D-5L scores at long-term follow-up were 84.9 (95% CI, 80.5–89.3) and 83.7 (IQR, 77.8–100.0) in the primary closure group, and 86.5 (95% CI, 80.6–92.4) and 91.7 (IQR, 76.5–100.0) in the mesh group. From baseline to 1-year follow-up, patients in the primary closure group revealed a relative increase of 3.9% in mean VAS and 2.2% in mean EQ-5D-5L scores, whereas the mesh group showed increases of 1.1% and 2.1%, respectively. Furthermore, a median increase from baseline to 1-year follow-up for the primary closure group of 6.3% was noted in the VAS and a decrease of 1.0% in the EQ-5D-5L scores, whereas the mesh group showed no difference in the VAS and an increase of 8.5% in the median EQ-5D-5L scores.

No statistically significant differences in VAS or EQ-5D-5L scores were observed between treatment arms over time based on repeated-measures beta-regression analyses (Table 2). Patients who experienced perineal complications demonstrated significantly lower VAS (–7.3%; 95% CI, –13.3% to –1.4%) and EQ-5D-5L (–12.4%; 95% CI, –18.5% to –6.2%) scores over time compared to those without such complications (Table 3).

**TABLE 2.** VAS and EQ-5D-5L scores over time in patients who underwent biological mesh closure compared with those who underwent primary perineal closure group

*Model*	*Intercept (estimate ± SE*)	*Time,^a^ estimate (95% CI*)	*Randomized to biological mesh, estimate (95% CI*)
VAS score	72.86 ± 1.91	0.021 (0.012 to 0.030), *p <* 0.001	–4.551 (–9.645 to 0.558), *p* = 0.081
EQ-5D-5L score	0.749 ± 0.021	0.000270 (0.000174 to 0.000364), *p <* 0.001	0.018 (–0.037 to 0.074), *p* = 0.520

EQ-5D-5L = EuroQoL five-dimension, five-level questionnaire; VAS = visual analog scale.

a Time measured in months since surgery.

**TABLE 3.** VAS and EQ-5D-5L scores over time in patients with perineal complications compared with those without such complications

*Model*	*Intercept (estimate ± SE*)	*Time,^a^ estimate (95% CI*)	*Perineal complication*, *estimate (95% CI*)	*Randomized to biological mesh, estimate (95% CI*)
VAS score	74.68 ± 2.014	0.021 (0.012 to 0.030), *p <* 0.001	–7.311 (–13.344 to –1.376), *p* = 0.017	–4.980 (–9.936 to –0.017), *p* = 0.051
EQ-5D-5L score	0.786 ± 0.016	0.000274 (0.000179 to 0.000369), *p <* 0.001	–0.124 (–0.185 to –0.062), *p <* 0.001	0.010 (–0.041 to 0.062), *p* = 0.705

EQ-5D-5L = EuroQoL five-dimension, five-level questionnaire; VAS = visual analog scale.

a Time measured in months since surgery.

### Combined Costs of the Intervention

**FIGURE 1.**
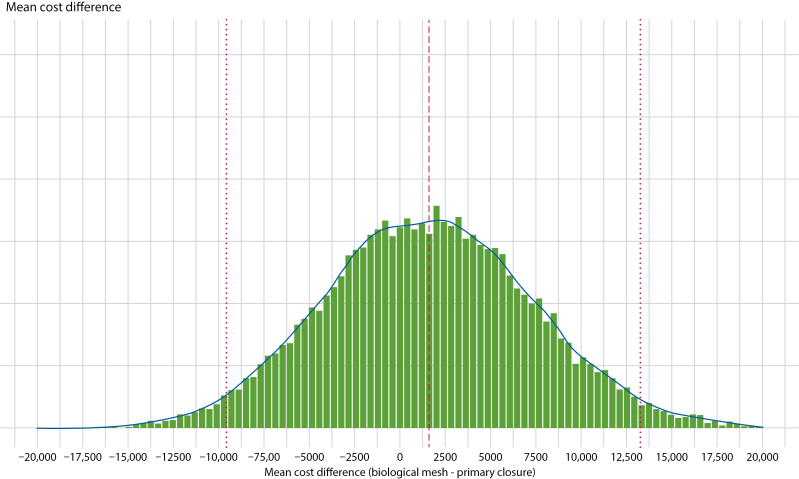
Histogram of the bootstrapped differences in costs between the randomization groups.

The mean total costs, incorporating procedural, posthospitalization, wound care, and rehospitalization expenses, were €56,232 (95% CI, 49,185–63,279) in the primary closure group and €57,991 (95% CI, 48,648–66,933) in the biological mesh group. After bootstrapped sampling, the Welch *t* test mean cost difference was €1571 (95% CI, –€9840 to €12,981) in favor of the primary closure group (*p* = 0.785; Fig. 1). In addition to the base-case analysis, we also performed a scenario analysis on a single data set including the mean of the 10 imputed values. Results are available in the Supplementary Files and are in line with the base-case results (see Supplemental Figure 1 at https://links.lww.com/DCR/C634).

### Sensitivity Analysis

Across the 1-way scenarios, the mean cost difference remained positive, indicating that the mesh strategy was more expensive for every tested parameter value. When varying the mesh price from 50% (€900) to 150% (€2700) of the base value, the mean cost difference ranged from €671 to €2471. When varying extra operating time from 50% (26 minutes) to 150% (78 minutes) while holding the mesh price constant, the cost difference ranged from €1282 to €1859. These results are illustrated in a tornado plot (see Supplemental Figure 2 at https://links.lww.com/DCR/C635). The 2-way sensitivity heatmap shows that across the full evaluated grid of mesh prices and extra OR time, the mesh strategy remained more costly. No breakeven contour (cost difference = 0) was observed within the parameter space, indicating that a 50% reduction in mesh price in combination with a 50% reduction in extra OR time did not result in cost savings for the mesh group (see Supplemental Figure 3 at https://links.lww.com/DCR/C636).

## DISCUSSION

This economic evaluation of perineal closure after eAPR using a biological mesh versus primary perineal wound closure demonstrates that the use of a biological mesh is associated with higher procedural and postoperative costs, which are not offset by significant improvements in required wound management, lowered incidence of perineal hernia repair, reduced costs, or more QALYs during a 5-year period.^11,12^ CIs of total costs overlapped and showed high heterogeneity. Therefore, no definitive evidence for superiority of treatment was found in the total population. Consequently, the routine use of biological mesh cannot be justified on a cost-effectiveness standpoint.

Perineal wound complications remain a major source of postoperative morbidity and significantly reduced HRQoL. In our study, patients with perineal morbidity reported significantly lower scores on both EQ-5D-5L and VAS, highlighting the burden of these events from both a clinical and patient perspective. These findings are consistent with previous reports linking perineal wound issues to persistent functional limitations and psychological distress.^3,5,16^ Importantly, our data showed similar HRQoL outcomes for both methods of perineal wound closure. Complications were the dominant determinant of reduced QoL, which suggests that efforts to optimize outcomes should focus on preventing morbidity.^3^

Although biological mesh reconstruction was associated with a reduction in reoperations for perineal hernia, the study was not powered to detect statistically significant differences for this outcome. These observations align with previous nonrandomized studies that reported relatively low perineal hernia rates after mesh reconstruction but acknowledged the need for larger trials.^10,17^ Importantly, this potential clinical benefit did not result in reduced overall health care utilization. In fact, the total mean costs per patient were €1571 higher in the mesh group, driven largely by higher index hospitalization and procedural costs. It should be noted, however, that the additional procedural costs partially reflect longer operative time, which may not translate directly into higher health care expenditures in practice. Bootstrapped cost-effectiveness analyses indicated a low probability that the biological mesh would be a cost-saving strategy.^14^ Furthermore, the sensitivity analyses confirmed that the base-case conclusion was robust to plausible variations in mesh price and additional operating time. Even when the mesh price was reduced by 50% or the extra OR time was reduced by 50%, the mesh strategy remained more expensive than primary closure. These findings indicate that neither substantial reductions in material cost nor modest gains in operative efficiency are sufficient to alter the overall economic conclusion.

Although routine use of biological mesh after eAPR cannot be justified on a cost-effectiveness standpoint, one might hypothesize that selective use in patients at particularly high risk could be cost-effective. This would apply mainly to more extensive resections, such as exenteration, where pelvic floor defects are larger and hernia risk is substantially elevated.^18^ In these settings, a biological mesh may offer structural support in addition to tissue flaps that are often needed for tensionless perineal closure and filling of perineal dead space. However, neither the BIOPEX trial nor the current literature provides any relevant evidence on the cost-effectiveness of a more tailored application of biological mesh reconstructions. Future trials should therefore focus on these high-risk subgroups, assessing both hernia incidence and long-term cost-effectiveness before any evidence-based recommendations can be made.^4,10^ Flap-based reconstructions remain important alternatives for managing large pelvic defects and filling dead space; however, comparative evidence directly comparing mesh and flap-based perineal closure strategies is limited. Ongoing trials and future subgroup-focused studies should evaluate clinical outcomes, resource use, and long-term HRQoL across mesh, flap, and hybrid approaches to better inform tailored reconstruction strategies.^16^

A key reason many surgeons continue to advocate for biological mesh is the belief in preventing perineal hernia, thereby reducing the need for reoperation and improving long-term QoL. Although a reduction in perineal hernia reoperations was observed in the BIOPEX trial, this did not translate into lower overall health care utilization or improved cost-effectiveness. As we have observed in daily practice, patients may not be offered perineal hernia repair. This may be due to limited expertise and the technical challenges of such a repair, although literature on this topic is lacking. Also, perineal hernia symptoms can be mild and the risks of reoperation in a previously operated and often radiated field might be considered to be relatively high, potentially contributing to negative advice regarding surgical repair. The low reoperation rates observed in our study may reflect both patient and surgeon barriers to hernia repair rather than a true absence of clinically significant hernias, highlighting the need for more detailed, long-term evaluations of hernia-related outcomes.

Our study addresses a gap in literature by combining clinical outcomes with long-term economic evaluation. Although prior studies, such as Baloch et al and Thomas et al, suggested clinical advantages of mesh reconstruction of the pelvic floor, robust cost data were lacking.^9,10^ The ongoing GRECCAR 9 trial may offer further insight.^16^ Future research should aim to identify patient subgroups most likely to benefit from mesh closure and assess the long-term impact of mesh use on hernia prevention and HRQoL.^10^ As results show great heterogeneity in costs, future trials should include power calculations that take this level of uncertainty in mind.

### Strengths and Limitations

Strengths of this study include the multicenter design in a real-world clinical setting with standardized follow-up and prospective economic data collection, elements that enhance its external validity. Nonetheless, the relatively small sample size constrained the power of subgroup analyses and limited precision in cost-effectiveness estimates. Furthermore, the low number of perineal hernia repairs raises questions about the health care system’s access to surgical repair and the experience of surgeons performing such procedures. Although multiple imputations addressed missing data methodologically, it also introduces uncertainty. In addition, the generalizability of findings may be affected by the use of a specific mesh type. Dutch centers operate under a social health insurance model with regulated tariffs, which may affect generalizability to other funding systems. Finally, health-related QoL was assessed only up to 12 months; thus, the impact of late-onset perineal hernias on patient-reported outcomes beyond 1 year could not be analyzed.

## CONCLUSIONS

Biological mesh reconstruction of the pelvic floor during perineal closure after eAPR for low rectal cancer does not significantly modify health care costs and does not show demonstrable improvements in patient-centered outcomes or QALY. Given these findings, routine use of biological mesh for pelvic floor reconstruction cannot currently be recommended from a cost-effectiveness standpoint. Future studies should assess patient-specific risk profiles for applying biological mesh after pelvic resections with certain pelvic floor defects.

## Supplementary Material
