# Supplementary material for: Cost-Effectiveness of Pelvic Floor Reconstruction Using Biological Mesh After Extralevator Abdominoperineal Resection for Rectal Cancer
Source: Dis Colon Rectum. 2026 Jun 15;69(9):2312–20. doi: 10.1097/DCR.0000000000004326 (PMC13465701; doi:10.1097/DCR.0000000000004326)
Supplement: Supplementary file 1 [file dcr-69-2312-s004.pdf]

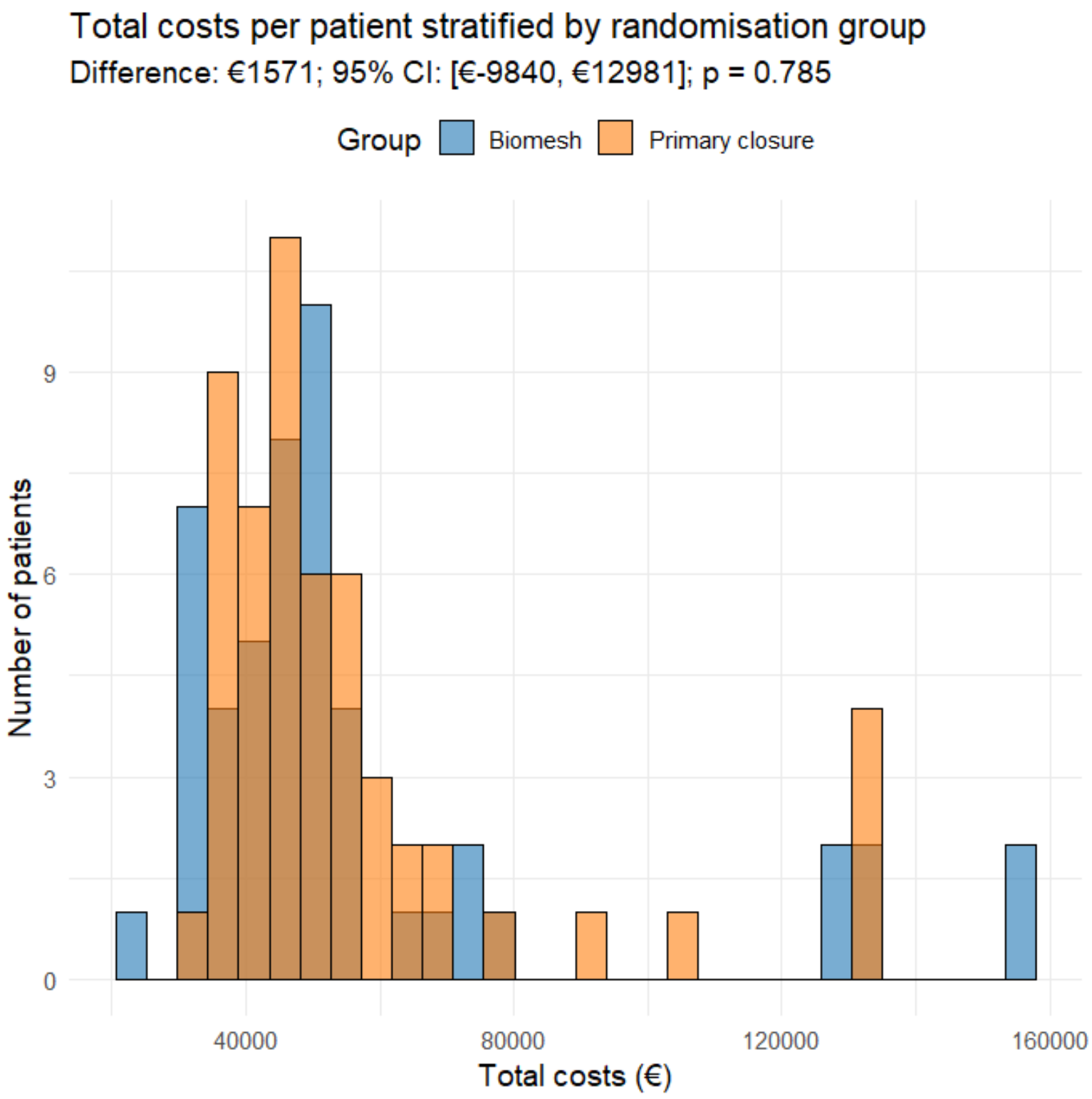
Total costs per patient stratified by randomisation group
Difference: €1571; 95% CI: [€-9840, €12981]; p = 0.785
Group
Biomesh
Primary closure
Number of patients
0
3
6
9
40000
80000
120000
160000
Total costs (€)

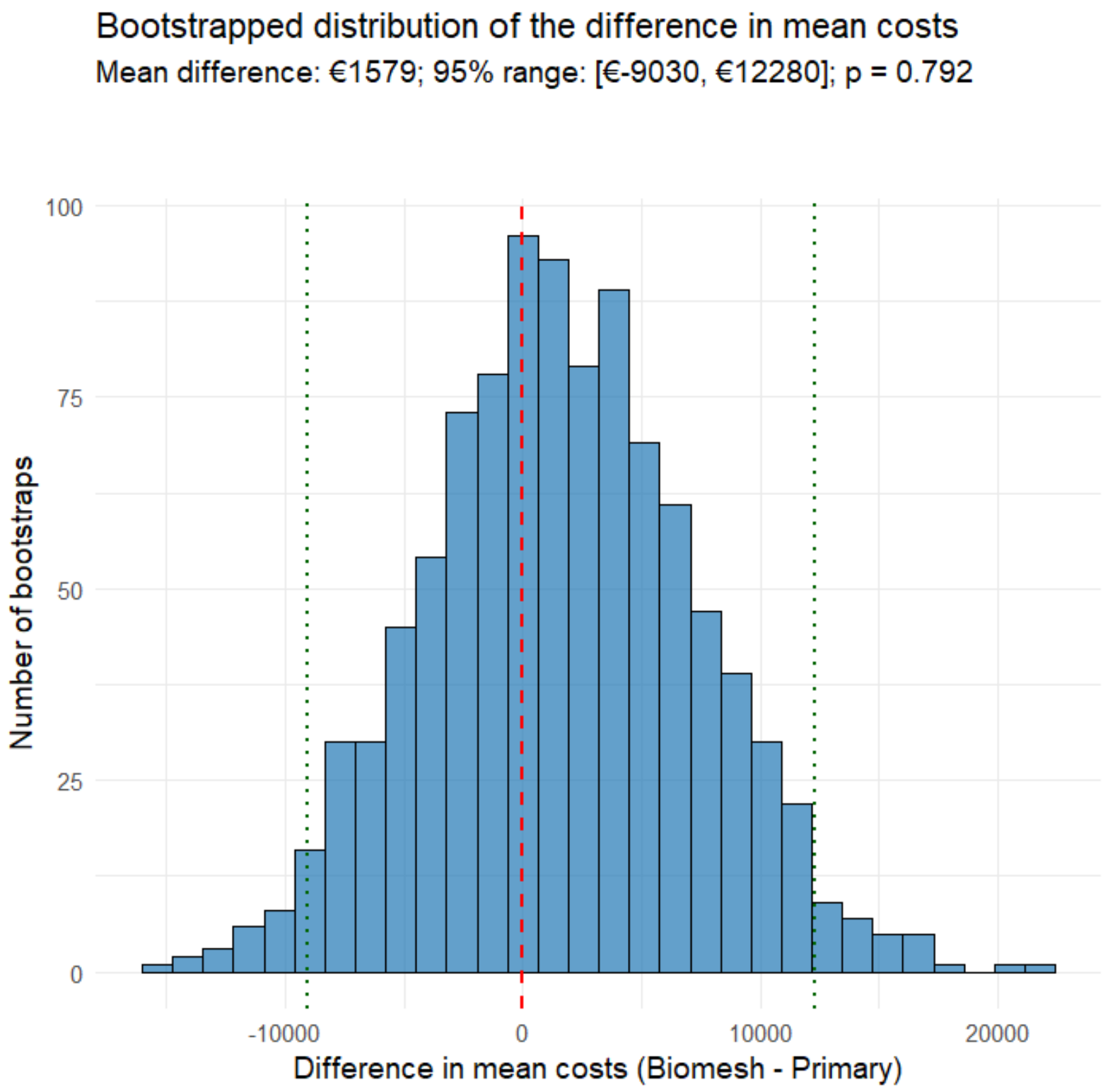
Bootstrapped distribution of the difference in mean costs
Mean difference: €1579; 95% range: [€-9030, €12280]; p = 0.792
Number of bootstraps
0
25
50
75
100
-10000
0
10000
20000
Difference in mean costs (Biomesh - Primary)
