# Supplementary material for: Cost-Effectiveness of Pelvic Floor Reconstruction Using Biological Mesh After Extralevator Abdominoperineal Resection for Rectal Cancer
Source: Dis Colon Rectum. 2026 Jun 15;69(9):2312–20. doi: 10.1097/DCR.0000000000004326 (PMC13465701; doi:10.1097/DCR.0000000000004326)
Supplement: Supplementary file 2 [file dcr-69-2312-s005.pdf]

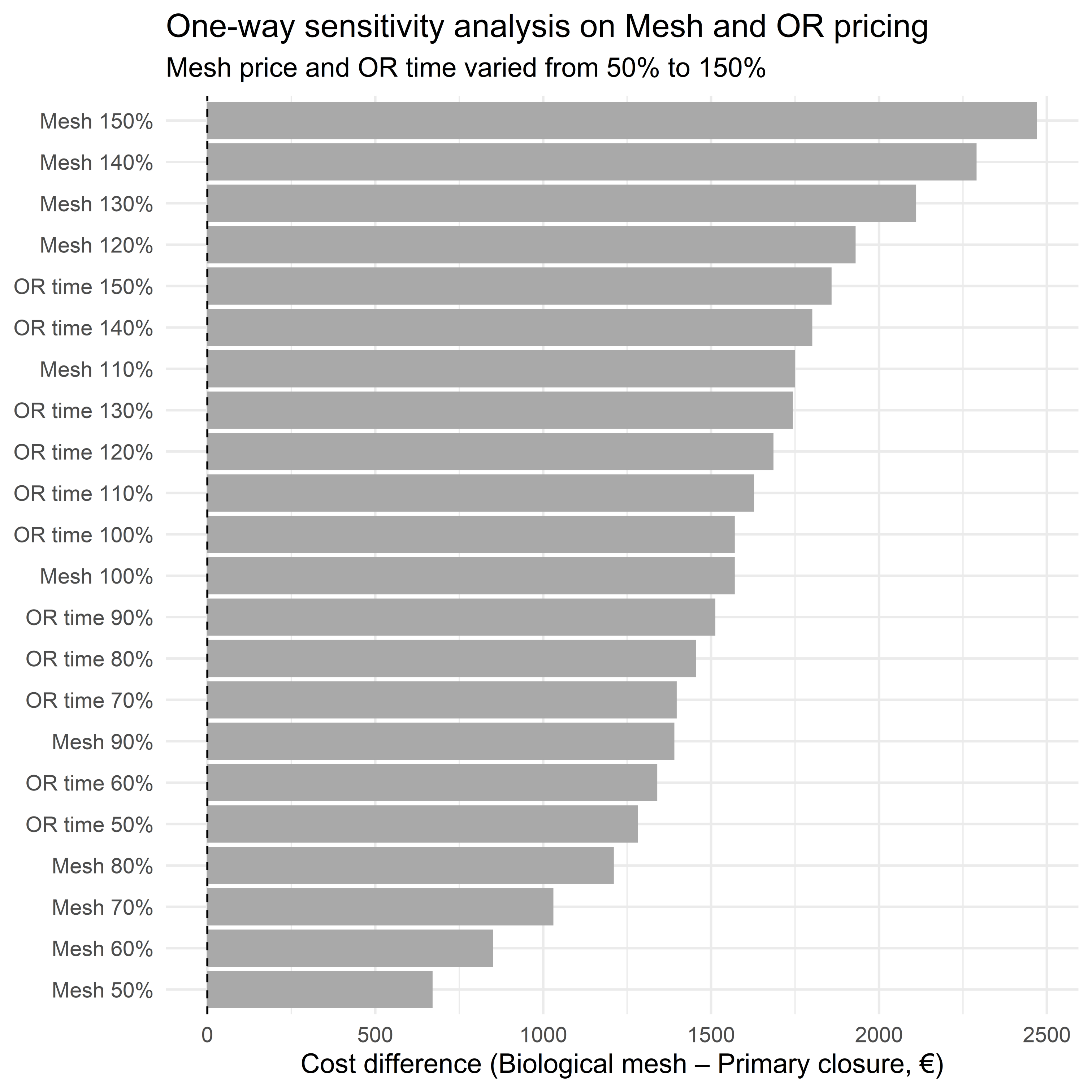
One-way sensitivity analysis on Mesh and OR pricing
Mesh price and OR time varied from 50% to 150%
Mesh 150%
Mesh 140%
Mesh 130%
Mesh 120%
OR time 150%
OR time 140%
Mesh 110%
OR time 130%
OR time 120%
OR time 110%
OR time 100%
Mesh 100%
OR time 90%
OR time 80%
OR time 70%
Mesh 90%
OR time 60%
OR time 50%
Mesh 80%
Mesh 70%
Mesh 60%
Mesh 50%
0
500
1000
1500
2000
2500
Cost difference (Biological mesh – Primary closure, €)
