# Supplementary material for: Cost-Effectiveness of Pelvic Floor Reconstruction Using Biological Mesh After Extralevator Abdominoperineal Resection for Rectal Cancer
Source: Dis Colon Rectum. 2026 Jun 15;69(9):2312–20. doi: 10.1097/DCR.0000000000004326 (PMC13465701; doi:10.1097/DCR.0000000000004326)
Supplement: Supplementary file 3 [file dcr-69-2312-s006.pdf]

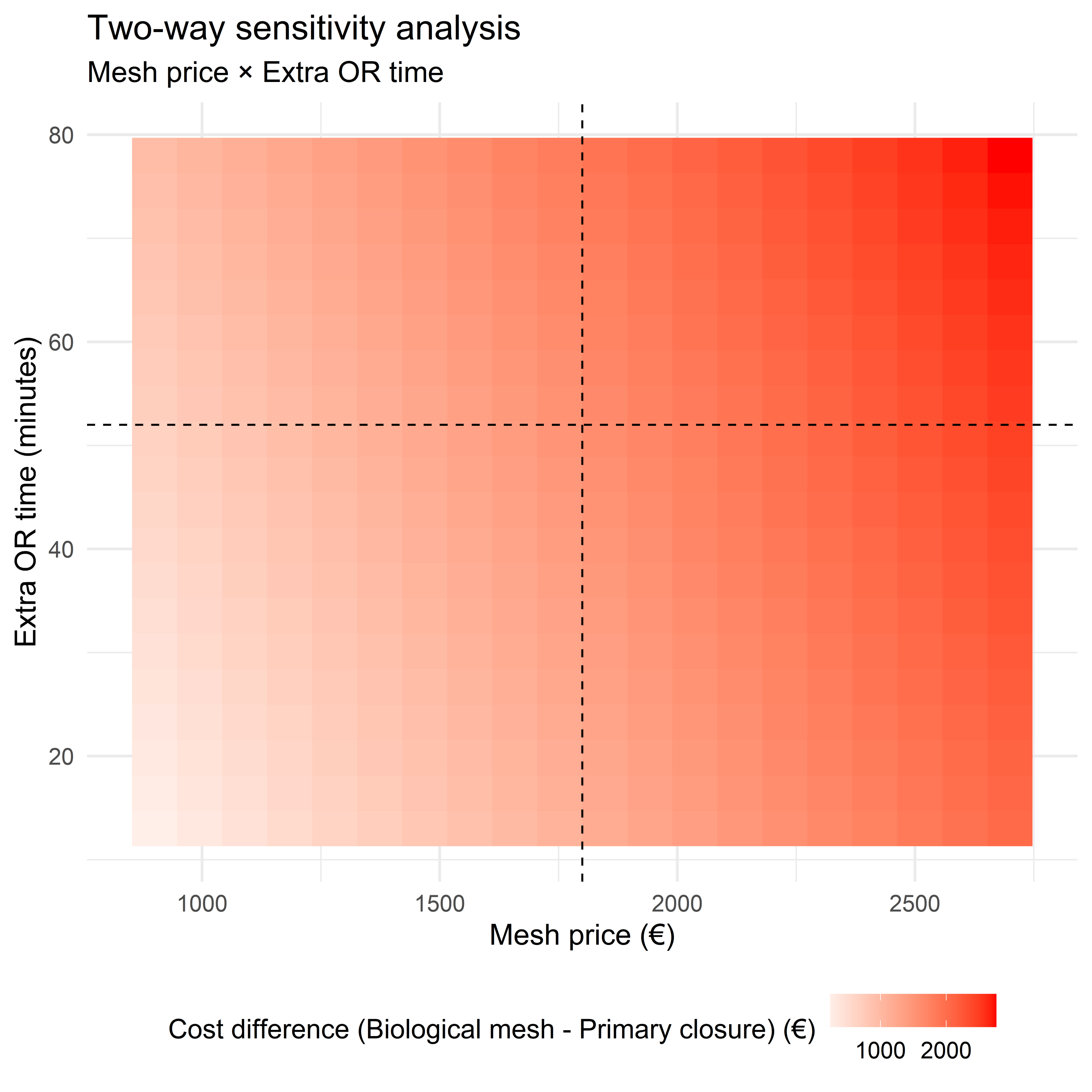
Two-way sensitivity analysis
Mesh price × Extra OR time
Extra OR time (minutes)
80
60
40
20
1000
1500
2000
2500
Mesh price (€)
Cost difference (Biological mesh - Primary closure) (€)
1000
2000
